# Characteristics and treatment of pediatric nasal foreign bodies with button batteries-A retrospective analysis of 176 cases

**DOI:** 10.1371/journal.pone.0309261

**Published:** 2024-08-29

**Authors:** Shang Yan, Guowei Chen, Nan Zeng, Chaobing Gao

**Affiliations:** 1 Department of Otorhinolaryngology Head and Neck Surgery, First Affiliated Hospital of Anhui Medical University, Hefei, Anhui, China; 2 Department of Otolaryngology, Shenzhen Children’s Hospital, Shenzhen, Guangdong, China; University Putra Malaysia, MALAYSIA

## Abstract

**Purpose:**

Button battery nasal impactions pose serious risks due to complications and the need for prompt removal, yet research on interventions remains limited due to its rare occurrence. To delineate the clinical manifestations of nasal foreign bodies associated with button batteries and to explore treatment approaches focused on minimizing the reliance on general anesthesia and surgical interventions.

**Methods:**

This study focuses on 176 cases of children who received treatment for nasal cavity button battery impactions. It encompasses various factors including age, gender, battery location, impaction duration, methods of extraction, and associated complications.

**Results:**

The incidence of nasal button battery cases among nasal foreign body instances was 1.16%, with a majority being males (60.23%) aged 1–5 years (98.29%). Utilizing a specially designed nasal foreign body hook and following established treatment protocols enabled the successful outpatient management of the majority of cases. Only 12 cases (6.82%) necessitated removal under general anesthesia due to management challenges in an outpatient setting. Furthermore, our findings indicated no linear correlation between the duration of battery retention and the risk of nasal septal perforation, which was observed in 31 cases (17.61%).

**Conclusion:**

Nasal foreign bodies caused by button batteries in children demand urgent attention due to their potentially grave outcomes. Our research is directed towards enhancing diagnostic and therapeutic strategies to bolster the success rates of outpatient removal, curtail the duration of foreign body retention, and diminish the reliance on general anesthesia.

## Introduction

Nasal foreign bodies are a prevalent issue in pediatric emergency care, especially among children under 5 years old, reflecting their innate curiosity and tendency to explore their environment by inserting objects into their nasal cavities, including toys and food particles [1–3]. Among these, button batteries pose a unique and significant risk, capable of causing severe chemical burns and tissue necrosis. Recognizing and understanding the dangers associated with nasal button batteries is critical for their effective management and the prevention of serious complications. Research into nasal button battery cases has often been hampered by the rarity of these incidents, leading to studies with relatively small sample sizes. Concurrently, there is a high proportion of cases requiring surgical intervention, with rates documented between 41.67% and 64.71% [4–6]. Importantly, previous studies have not adequately emphasized the need for quick, outpatient removal of button batteries—a strategy that could mitigate the need for general anesthesia, shorten the duration of exposure to battery-related injuries, and optimize healthcare resource utilization. This study aims to provide comprehensive insights into nasal button battery foreign bodies, facilitating precise treatment approaches and enhancing the success of outpatient removal.

## Materials and methods

A retrospective analysis covering the period from August 2014 to July 2021 identified 176 cases of children with nasal button battery insertions among 15,203 nasal foreign body medical records from Shenzhen Children’s Hospital. Data were accessed for research purposes between December 2021 and May 2022. The study gathered demographic and clinical data, such as gender, age, reasons for impaction, battery location, duration of impaction, methods of extraction, and any associated complications.

**Fig 1:**
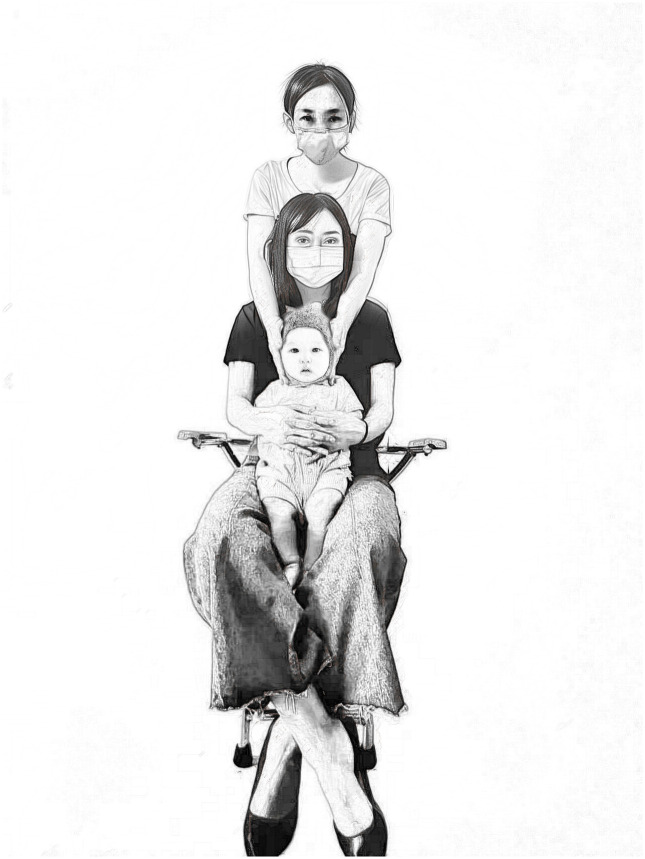
Illustration of parental assistance in stabilizing the child’s position.

Before beginning the removal process of a button battery nasal foreign body, it’s essential to first ensure the child is calm and cooperative. We engage parents to assist in stabilizing the child, aiming to prevent any sudden movements or distress that could complicate the procedure (Fig 1). Next, we clear the nasal cavity of any secretions to provide a clear view and minimize discomfort.

**Fig 2:**
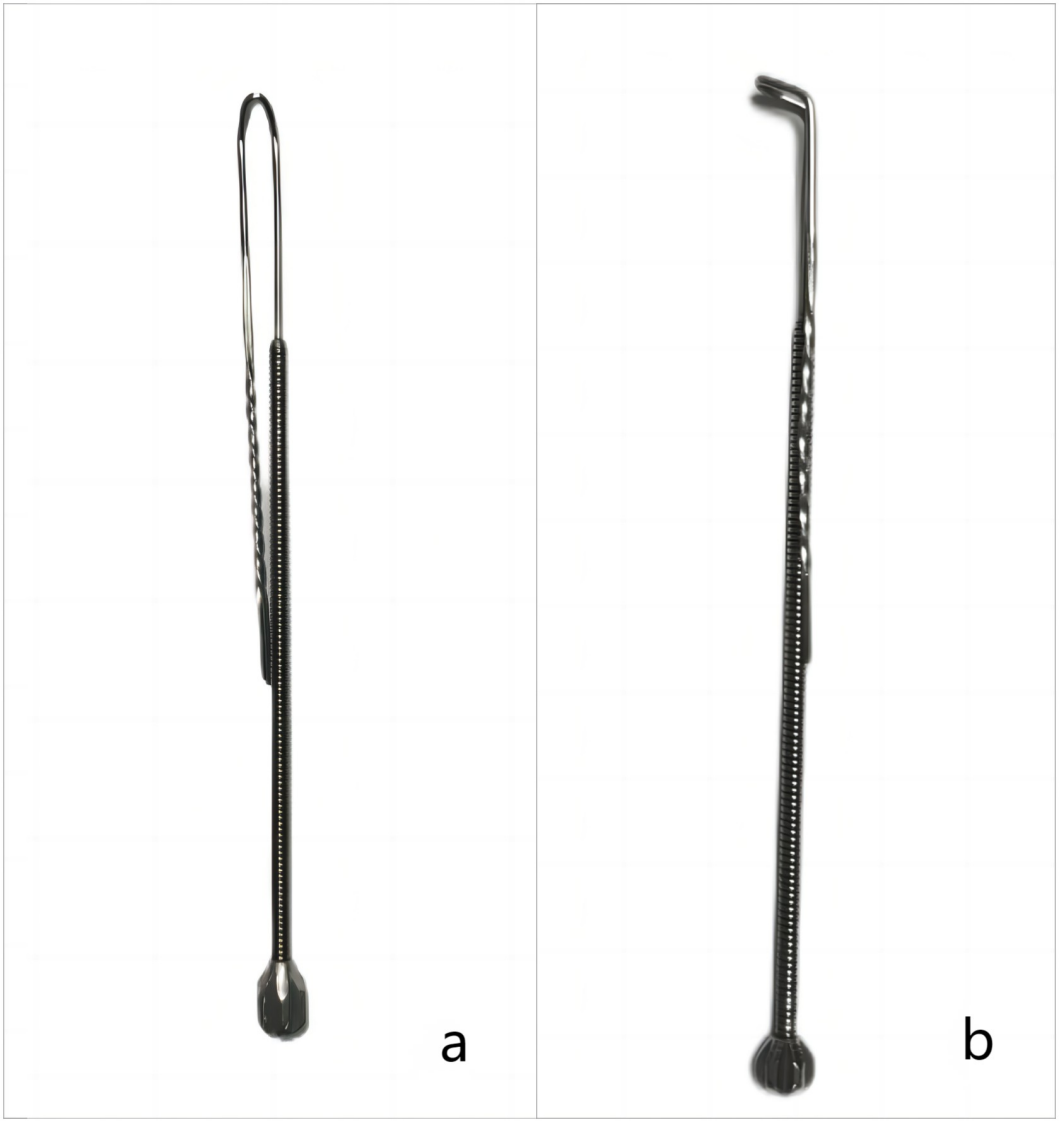
a) Frontal view of nasal foreign body hook. b) Lateral view of nasal foreign body hook.

For illumination and examination, we utilize an LED headlamp along with anterior rhinoscopy. If the button battery is visible, we attempt its removal using a specially designed nasal foreign body hook (Fig 2). Its upper part is constructed from smooth metal wire, forming a hooked shape at the end, while the lower part consists of a thicker handle. The distinctive feature of this hook lies in the smooth surface of the metal wire and the hollow U-shape of the head, providing sufficient elasticity and minimizing inadvertent mucosal damage during manipulation. The hooked structure at the head ensures a substantial contact area, enabling the maintenance of considerable force even when dealing with cases of severe adhesion.

In scenarios where the button battery is not directly visible due to swelling or excessive secretions, but its presence is strongly suspected, we proceed with parental consent to attempt removal. The nasal foreign body hook is inserted through the anterior nostril, navigated through the middle meatus to the vicinity of the posterior nostril, then directed downward to the nasal floor before being gently pulled, attempting to extract the deeper-seated foreign object.

If a foreign body was not detected and metallic foreign body impaction was suspected, anteroposterior and lateral views of the nose were obtained through plain radiographs for precise localization. Upon confirming the presence of a metallic foreign body via X-ray, we once again sought parental cooperation for a final attempt at removal in the outpatient setting. If these attempts remained unsuccessful, and the foreign body could not be extracted through outpatient procedures, we proceeded with prompt surgical intervention. This involved general anesthesia and nasal endoscopy to ensure the safe and complete extraction of the nasal foreign body.

This study was conducted in compliance with the principles of the Declaration of Helsinki for Biomedical Research Involving Human Subjects and was approved by the Ethical Review Board of Shenzhen Children’s Hospital (protocol number 2021028). Written informed consent was obtained from the parents and/or legal guardians of each child participant. Authors did not have access to information that could identify individual participants during or after data collection.

## Results

### Demographic data

During the seven-year study period from 2014 to 2021, a total of 176 children with nasal button battery impaction were treated in the Department of Otolaryngology. The average age was 2.97 years (±2.16 SD), with a range of 1 to 8 years. Among these children, 173 (98.29%) were aged 1 to 5 years. The gender distribution was 106 boys (60.23%) and 70 girls (39.77%). The supplementary age and gender distribution data is shown in S1 Fig.

### Patient presentation

All patients presented with complaints of self-inserted foreign objects in their nasal cavities, which were subsequently noticed by their parents or caregivers. Among the 176 children, 162 cases (92.05%) involved nasal button batteries identified within 24 hours of insertion. Twelve children (6.81%) had button batteries identified within one week but more than one day after insertion, while two children (1.13%) had an uncertain timeframe.

### Battery location and size

Nasal button batteries were more frequently found in the right nasal cavity (106 cases, 60.23%) compared to the left (70 cases, 39.77%). Most batteries (168 cases, 95.45%) had a diameter of 6-12mm, while 8 cases (4.55%) involved batteries with a diameter of 12-16mm.

### Battery removal

A total of 148 cases (84.09%) were located between the posterior portion of the nasal vestibule and the anteroinferior portion of the nasal cavity. These batteries were removed using standard equipment in the consultation room. However, 28 cases (15.91%) were in the medioposterior region of the nasal cavity. Despite exhaustive outpatient attempts, 12 cases required removal under general anesthesia. Four cases (2.27%) were transferred to our institution after unsuccessful extraction attempts at other facilities, necessitating general anesthesia for removal.

### Comorbidities

Following button battery removal, nasal endoscopy was performed, revealing several comorbidities. Rhinitis was observed in 54 cases (30.68%), while rhinosinusitis was present in 60 cases (34.09%). Adenoid hypertrophy was found in 37 cases (21.02%), and a combination of adenoid hypertrophy and rhinosinusitis was observed in 16 cases (9.09%). Additionally, 13 cases (7.39%) showed both adenoid hypertrophy and rhinitis. Supplementary comorbidities and complications are summarized in S2 Fig.

### Complications

Corrosive injuries to the nasal mucosa led to various complications. Epistaxis was observed in 164 cases (93.18%) during battery removal. Nasal adhesion occurred in 3 instances (1.70%), and septal perforations were identified in 31 cases (17.61%) during follow-up examinations. In a case involving a one-year-old child with a button battery lodged for an hour, a septal perforation was observed immediately after extraction. Among 12 patients with batteries lodged for more than 24 hours, only 2 exhibited septal perforations at follow-up. No septal perforations were detected at follow-up in two children with uncertain insertion durations. The risk of septal perforation is influenced by multiple factors, and there does not appear to be a simple linear relationship between the duration of battery presence and the risk of septal perforation. The demographic and clinical data of the nasal button battery impaction cases are summarized in Table 1.

**Table 1:** Characteristic of button battery nasal foreign bodies children.

Characteristic	Number	Percent (%)
1. Gender		
boys	106	60.22
girls	70	39.77
2. Age (years)		
1	11	6.25
2	43	24.43
3	80	45.45
4	30	17.05
5	9	5.11
6–8	3	1.70
3. The side of foreign bodies		
the left	70	39.77
the right	106	60.23
4. The location of foreign bodies in nasal cavity		
the anteroinferior portion	148	84.09
the medioposterior portion vity cavity	28	15.91
5. The duration of foreign bodies		
≤1day	162	92.05
>1day to ≤1week	12	6.81
uncertain	2	1.13
6. The removal methods of foreign bodies		
remove in the outpatient and emergency department	164	93.18
remove under general anesthesia in hospital	12	6.81
7. Comorbidities		
rhinitis	54	30.68
sinusitis	60	34.09
adenoid hypertrophy	37	21.02
8. The complications of foreign bodies		
epistaxis	164	93.18
nasal adhesion	3	1.70
nasal septum perforation	31	17.61

## Discussion

**Fig 3:**
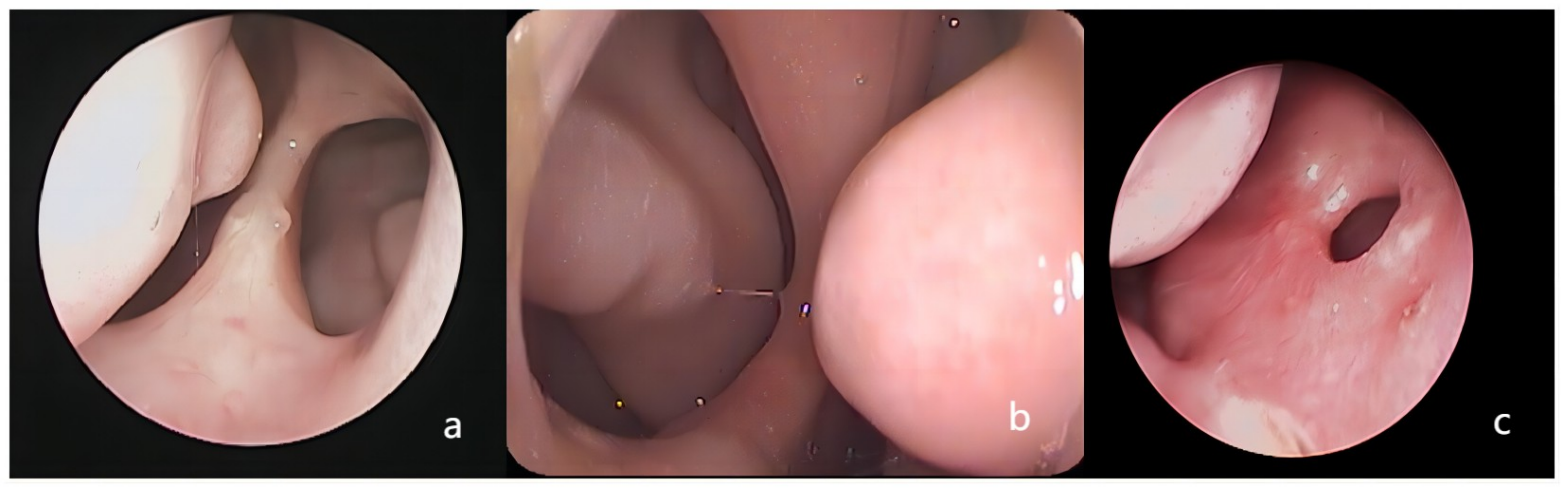
a, b, c) nasal septum perforation resulting from button battery insertion in the nasal cavity.

Nasal button battery impaction in children is a rarely encountered condition that can result in significant complications [1–3, 7]. There are four mechanisms that contribute to tissue injury caused by nasal button batteries. First, the corrosive electrolyte solution can leak and be absorbed by the nasal mucosa, resulting in deep tissue damage [8]. Second, the direct current can cause a burn to the nasal mucosa, leading to the exudation of tissue fluids and the creation of a moist environment that further promotes electrolyte solution leakage [9]. Third, the pressure exerted by the battery, along with the previous two factors, can contribute to liquefactive necrosis of the surrounding tissues [9]. A previous study has suggested that direct current stimulation may be the major factor contributing to tissue damage [10]. Due to the rare occurrence of nasal button battery impaction, many parents remain unaware of this issue. Furthermore, medical professionals might lack the required expertise to manage such cases adequately or possess appropriate instruments for removing nasal foreign bodies. Consequently, there can be delays in the removal of button batteries, potentially leading to the object being pushed deeper into the nasal cavity. This scenario might require surgical intervention under general anesthesia with nasal endoscopy to retrieve the battery. Ultimately, these circumstances can result in prolonged damage to the nasal mucosa and even nasal septal perforation (Fig 3). The growing usage of button batteries in electronic devices like toys, remote controls, and hearing aids has contributed to a surge in cases of nasal button battery impaction [9, 11]. Predominantly, these cases involve children aged one to five, likely due to their innate curiosity [3, 12]. Children often insert button batteries into their nasal cavities themselves, attracted by the batteries’ shiny appearance, and accidental insertion can occur during play when caregivers are momentarily distracted [13–15]. When a child’s nose remains uncomfortable for an extended period, they are more likely to insert foreign objects into the nasal cavity. Following the removal of button batteries, the study revealed a notable prevalence of sinonasal diseases and adenoid hypertrophy, with respective proportions of rhinitis (30.68%), sinusitis (34.09%), and adenoid hypertrophy (21.02%). This presence of button batteries appeared to correlate with a higher occurrence of these sinonasal conditions compared to reported incidence rates in Chinese children, which were 10.80–21.09% for rhinitis, 6.37% for sinusitis, and 4.8% for adenoid hypertrophy [16–19]. Moreover, the study found a higher incidence of nasal foreign body impaction in males, consistent with previous investigations [15, 20–23]. These differences in incidence rates between sexes may be influenced by a combination of physiological and psychological factors, parenting styles, and the local sex ratio [4, 24].

When accidents involving the insertion of button batteries into the nasal cavity occur, the most critical action is to remove the foreign object as swiftly and safely as possible. Therefore, in our hospital, children with nasal cavity button batteries are given priority for consultation. Our treatment protocol is centered on the extraction of the object in an outpatient setting whenever possible, with the objectives of minimizing the necessity for surgical intervention and reducing the duration of button battery retention within the nasal cavity.

Initially, it involves gaining the cooperation of the parents, calming the child, and securely positioning the patient to prevent any sudden movements during the procedure that could lead to secondary injuries. Clear visibility is also of paramount importance. Utilizing an LED headlamp can provide enhanced illumination, as the insertion of button batteries into the nasal cavity often results in the leakage of a dark brown electrolyte, which mixes with nasal secretions. Before commencing the operation, it is necessary to thoroughly clean the area with a suction device to ensure clear visibility.

When the patient’s history clearly indicates the insertion of a button battery into a child’s nasal cavity, or if a dark brown electrolyte along with bubbles is visible in one of the anterior nostrils, these are strong indications of a button battery lodged within the nasal cavity. Even if the nasal mucosa is significantly swollen or the foreign object is too deep to be visually detected, it is crucial to promptly obtain the cooperation of the child’s parents and swiftly attempt to extract the foreign object using a retrieval hook. During the extraction process, if the retrieval hook encounters a hard object, it confirms the diagnosis of a nasal foreign body, negating the immediate need for imaging studies. This is because, in the early stages of a button battery being lodged in the nasal cavity, every minute can significantly exacerbate the extent of nasal injury to the child.

The importance of safe and reliable tools cannot be overstated. We have experimented with numerous tools, finding that most foreign body hooks are overly rigid and lack the necessary flexibility. This rigidity has led physicians to hesitate in applying sufficient force during procedures, fearing potential damage to the nasal structures. Additionally, some hooks do not have the optimal angle or are too smooth at the tip, failing to provide the necessary grip to remove button batteries that are tightly adhered to the tissue. The foreign body hooks we utilize, however, are designed with a smooth surface and moderate elasticity, allowing for the use of greater force without significant concern for causing severe damage to the nasal tissue structures, typically resulting in only minimal nasal mucosal bleeding.

**Fig 4:**
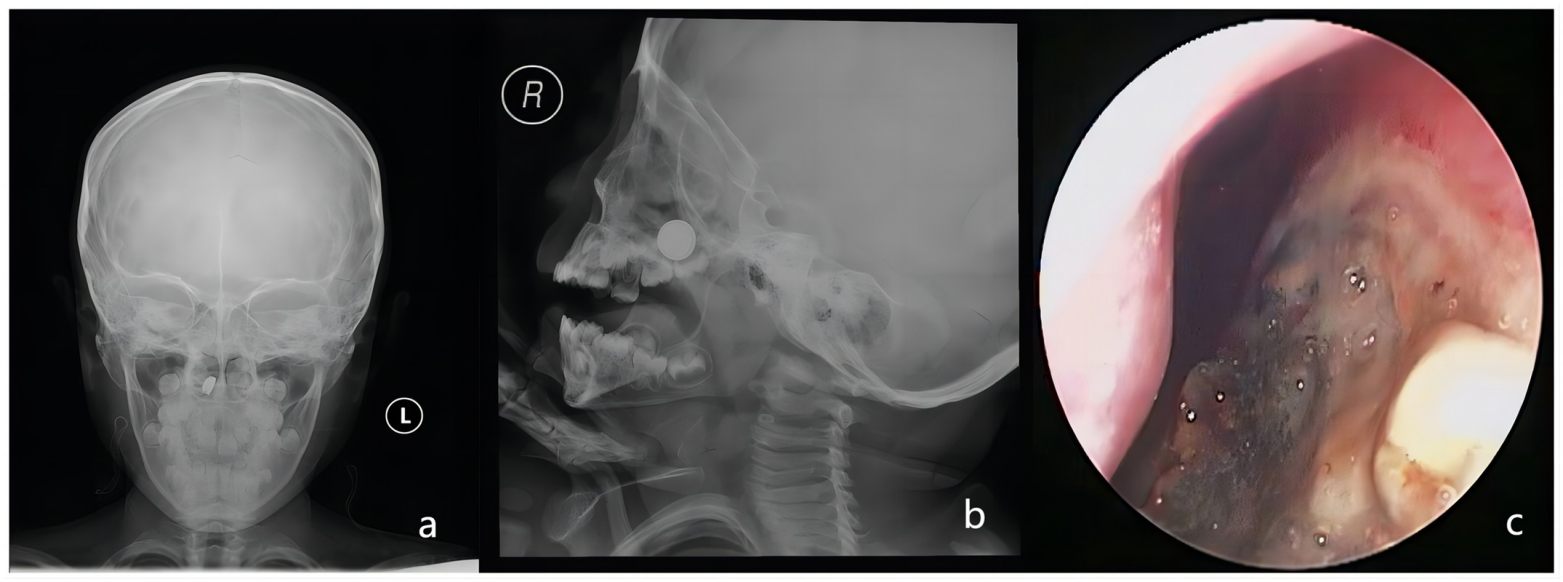
a) A step-off at the separation between the anode and cathode on the anteroposterior view. b) A double ring or halo on the lateral view. c) The nasal mucosa was significantly swollen, eroded, and necrotic (dark gray) on the surface.

In cases where a foreign body was not found or suspicion for a metal foreign body was high, a plain nasal radiograph was ordered, as recommended by current evidence [25–27]. This imaging method not only locates the button battery but also distinguishes it from other metal foreign bodies, given its unique bilaminar structure, with a step-off at the separation between the anode and cathode on the anteroposterior view (Fig 4A) and a double ring or halo on the lateral view (Fig 4B).

Upon confirming the diagnosis and locating the button battery, we engage in communication with the parents and, upon obtaining their consent, suggest one final attempt to remove the foreign object in the outpatient setting. At this juncture, the operating physician must rely on their experience to swiftly locate the button battery with the foreign body hook, even without visual aid and amidst the child’s distress. The physician extends the hook beyond the battery, aiming for its extraction. This represents one of the most challenging scenarios. Should all attempts fail to remove the object, we must immediately arrange for emergency surgery to extract the button battery from the child’s nasal cavity [28]. It’s important to note that uncooperative children may also necessitate general anesthesia, and inexperienced otolaryngologists could inadvertently push the button battery deeper into the nose, leading to unsuccessful removal attempts [29]. In our study, four children experienced failed removal attempts in other hospitals and required button battery removal under general anesthesia.

Following button battery removal, the nasal mucosa exhibited varying degrees of corrosive injuries. Mild cases showed swollen, slightly congested, and yellow surface mucosa, while severe cases displayed significantly swollen, eroded, and necrotic (dark gray appearance) surface mucosa, sometimes affecting the contralateral mucosa of the nasal septum (Fig 4C). We also observed a rapid release of dark brown heavy metal substances from button batteries within the nasal environment. In one case, a 3-year-old male patient’s button battery was removed from the nasal cavity after 30 minutes of insertion. However, we still noted the leakage of dark brown material from the negative pole edge of the battery. Additionally, mucosal damage on the negative pole side of the battery was often more severe. Among the 31 patients with nasal septal perforation, 20 cases exhibited direct contact of the negative pole side with the nasal septum. In the cohort of patients with button batteries inserted in the nasal cavity for less than a day, 29 cases of nasal septal perforation were identified. Conversely, among the twelve patients who retained the batteries for more than a day, septal perforations were observed in only two cases during follow-up. Furthermore, no septal perforations were detected in two instances where the duration of battery insertion was indeterminate. These two cases, with unknown battery insertion times, likely involved button batteries that were completely discharged. This could explain why the children did not experience severe discomfort, why the parents remained unaware, and consequently, why no nasal septal perforations occurred. Hence, there is no simple linear relationship between the duration of button battery retention in the nasal cavity and the occurrence of nasal septal perforation. However, this does not imply that button batteries inserted into the nasal cavity can remain without being removed as early as possible. The extent of nasal damage is related to the location, duration, remaining charge, capacitance of the embedded battery [30], and we speculate that the duration of battery retention in the nasal cavity and the degree of nasal damage may have a linear relationship, albeit a possibly very short one, even down to minutes. After a certain period, the duration of battery retention in the nasal cavity will significantly decrease the degree of nasal damage and may even approach zero. The incidence of complications depends on factors such as battery location, duration of impaction, remaining voltage and capacitance, cooperation of caregivers and children, and the experience and expertise of the otolaryngologist performing the removal [31].

Following our concerted efforts, among the 176 cases of children with button batteries lodged within, only 12 required the removal of the foreign objects under general anesthesia in the operating room, resulting in a surgical intervention rate of 6.82%. This figure is significantly lower than the previously reported rates of 41.67% to 64.71% in the literature [4–6]. Additionally, the incidence of nasal septal perforation observed during follow-up in our cohort was relatively low, with 31 cases of nasal septal perforation, accounting for 17.61%, compared to the previously reported range of 16.67% to 23.53% in the literature [4–6]. Notably, among these, three were very minor and suspected perforations, all of which completely healed within three months after the removal of the button batteries. Prompt removal of the button battery in an outpatient setting positively contributes to reducing the likelihood of nasal septal perforation. However, given the multitude of factors that can lead to septal perforation, the reduction in the incidence of perforation in this study is not considered significant.

Button batteries carry a higher risk and severity of complications compared to other nasal foreign bodies, with epistaxis and sinonasal infections being primary concerns [15, 20, 21, 32]. During the removal of button batteries, epistaxis was observed in 164 cases (93.18%). For mild cases, applying finger pressure was sufficient to control the bleeding, while serious cases required the use of xylometazoline spray. Our study employed various interventions to promote the recovery of the nasal mucosa from corrosive injuries following removal, including nasal lavage, recombinant human epidermal growth factor spray, glucocorticoid nasal spray, and oral antibiotics. Sinonasal diseases were managed with treatments such as nasal lavage and/or saline nasal spray, glucocorticoid nasal spray, and/or oral antibiotics [33]. The duration of the recovery period varied depending on the severity of injuries and the occurrence of sinonasal infection.

In this study, button batteries of various sizes were removed from the nasal cavity. Most cases (95.45%) involved button batteries measuring between 6 mm to 12 mm in diameter, while larger batteries up to 16 mm in diameter were found in 4.55% of cases. Even small button batteries can cause significant damage if left in the nasal cavity for a prolonged period, leading to tissue necrosis and other complications. Commonly found objects such as remote controls, toys, watches, calculators, and alarm clocks are the sources of most 6-12mm button batteries children come into contact with. However, avoiding these batteries may not be practical as larger button batteries (16-20mm) are also commonly present in household objects and can lead to esophageal impaction. Educating parents and caregivers about the dangers of button batteries and closely supervising children when they are playing with objects containing these batteries is important. Additionally, if a child is unable to insert the battery into their nasal cavity, they may attempt to insert it into their mouth. Button batteries pose severe risks if swallowed or lodged in the pharynx or windpipe. Endoscopic procedures, such as esophagoscopy or gastroscopy, can remove foreign bodies from the esophagus or stomach. For significant corrosion damage in the esophagus, a nasogastric tube can repair the mucosa, while gastric corrosion can be treated with anti-gastric acid drugs and protective agents. If the foreign body is in the intestine and not obstructing, it may pass through naturally, but surgical intervention may be necessary if it becomes stuck. If a button battery enters the trachea, immediate medical attention is crucial, and bronchoscopy can remove the foreign body to prevent respiratory harm. Thankfully, in the study of 176 cases of nasal button battery impaction in children, no cases were reported where the battery detached into the digestive tract or airway. However, it is essential to remain vigilant and seek immediate medical attention if a child inserts a button battery into their nasal cavity or swallows one.

Given the serious risks associated with nasal button battery impactions, future research should focus on developing preventive measures and educational strategies to reduce the incidence of such cases. One key area is the research and development of safer battery designs that minimize the likelihood of severe tissue damage. Strengthening safety education for parents and caregivers about the dangers of button batteries and the importance of keeping them out of children’s reach is also crucial. Additionally, it is important to develop and disseminate standardized protocols for the rapid and effective management of nasal foreign bodies in pediatric patients. These protocols should be made available to healthcare providers, including those in emergency departments and primary care settings.

Limitations of this study, such as its focus on a single institution and its retrospective design, must be acknowledged. Continuous research is essential for further enhancing safety and prevention measures.

## Conclusion

This article presents a retrospective analysis of data from 176 nasal cavity button battery cases treated over the past seven years, detailing our experiences. Compared to previous reports, we observed a notably lower proportion of cases requiring general anesthesia for surgery, as well as a lower incidence of postoperative nasal septal perforation.

## Supporting information

S1 Dataset Raw data for nasal button battery impaction in children. This dataset contains the raw data used in the study, provided in both.sav and.xlsx formats.
(XLS)

S1 Fig Age and gender distribution of patients. This figure shows the age and gender distribution of the 176 children treated for nasal button battery impaction.
(TIF)

S2 Fig Observed comorbidities and complications. This figure summarizes the comorbidities and complications observed in the patients after the removal of the nasal button batteries.
(TIF)
