# Supplementary figures and images for: Characteristics and treatment of pediatric nasal foreign bodies with button batteries-A retrospective analysis of 176 cases
Source: PLoS One. 2024 Aug 29;19(8):e0309261. doi: 10.1371/journal.pone.0309261 (PMC11361442; doi:10.1371/journal.pone.0309261)

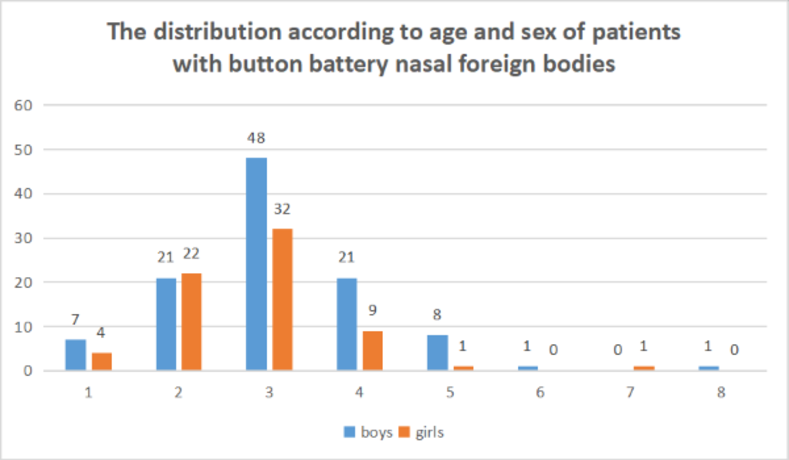

Supplement: S1 Fig — This figure shows the age and gender distribution of the 176 children treated for nasal button battery impaction. (TIF) [file pone.0309261.s002.tif]

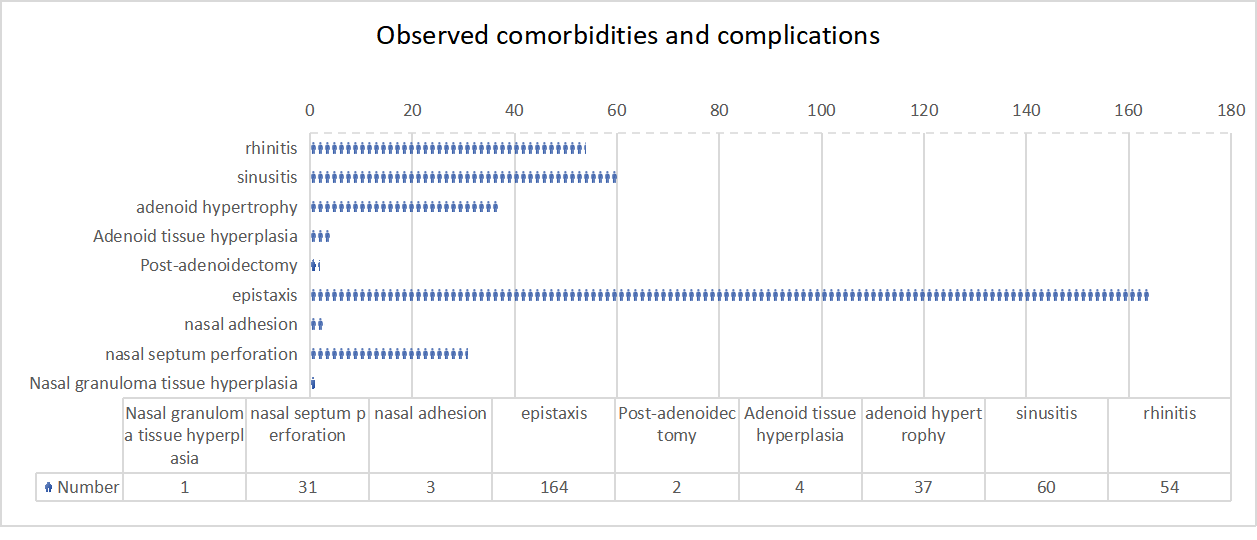

Supplement: S2 Fig — This figure summarizes the comorbidities and complications observed in the patients after the removal of the nasal button batteries. (TIF) [file pone.0309261.s003.tif]
